# Use of the emergency department for less-urgent care among type 2 diabetics under a disease management program

**DOI:** 10.1186/1472-6963-9-223

**Published:** 2009-12-07

**Authors:** Shang-Jyh Chiou, Claudia Campbell, Ronald Horswell, Leann Myers, Richard Culbertson

**Affiliations:** 1 Department of Health Care Administration, College of Health Science, Asia University, 500, Lioufeng Road, Wufeng, Taichung County 41354, Taiwan; 2 Department of Health Systems Management, Tulane University School of Public Health and Tropical Medicine, 1440 Canal Street, Suite 1900, New Orleans, LA 70112, USA; 3 Department of Biostatistics, Tulane University School of Public Health and Tropical Medicine, 1440 Canal Street, Suite 2000, New Orleans, LA 70112, USA; 4 Louisiana State University Health Sciences Center, Health Care Services Division, P.O. Box 91308, Baton Rouge, LA 70821, USA

## Abstract

**Background:**

This study analyzed the likelihood of less-urgent emergency department (ED) visits among type 2 diabetic patients receiving care under a diabetes disease management (DM) program offered by the Louisiana State University Health Care Services Division (LSU HCSD).

**Methods:**

All ED and outpatient clinic visits made by 6,412 type 2 diabetic patients from 1999 to 2006 were extracted from the LSU HCSD Disease Management (DM) Evaluation Database. Patient ED visits were classified as either urgent or less-urgent, and the likelihood of a less-urgent ED visit was compared with outpatient clinic visits using the Generalized Estimating Equation methodology for binary response to time-dependent variables.

**Results:**

Patients who adhered to regular clinic visit schedules dictated by the DM program were less likely to use the ED for less urgent care with odds ratio of 0.1585. Insured patients had 1.13 to 1.70 greater odds of a less-urgent ED visit than those who were uninsured. Patients with better-managed glycated hemoglobin (A1c or HbA1c) levels were 82 times less likely to use less-urgent ED visits. Furthermore, being older, Caucasian, or a longer participant in the DM program had a modestly lower likelihood of less-urgent ED visits. The patient's Charlson Comorbidity Index (CCI), gender, prior hospitalization, and the admitting facility showed no effect.

**Conclusion:**

Patients adhering to the DM visit guidelines were less likely to use the ED for less-urgent problems. Maintaining normal A1c levels for their diabetes also has the positive impact to reduce less-urgent ED usages. It suggests that successful DM programs may reduce inappropriate ED use. In contrast to expectations, uninsured patients were less likely to use the ED for less-urgent care. Patients in the DM program with Medicaid coverage were 1.3 times more likely to seek care in the ED for non-emergencies while commercially insured patients were nearly 1.7 times more likely to do so. Further research to understand inappropriate ED use among insured patients is needed. We suggest providing visit reminders, a call centre, or case managers to reduce the likelihood of less-urgent ED visit use among DM patients. By reducing the likelihood of unnecessary ED visits, successful DM programs can improve patient care.

## Background

According to the Centres for Disease Control and Prevention (CDC) [1], chronic diseases such as diabetes, cardiovascular disease, asthma and cancer are the most common and costly health problems in America with an annual cost totalling $174 billion in 2007 [2]. Chronic diseases have been the leading causes of death and disability in the United States for the past decade. In 2005, diabetes was the sixth most common disease in the U.S., and over 14.6 million Americans suffered from diabetes and diabetes-related comorbidities, such as hypertension, stroke or infections of the kidney. However, studies have concluded that type 2 diabetes is preventable, and patients with the disease can have a higher quality of life without suffering the chronic situation of high health care expenditures, if they follow their physician's advice on diet, medicines, and lifestyle behaviours [3-5]. Disease management (DM) is a systematic approach to identify the population at risk for specific diseases, especially chronic diseases, and to intervene with a program of care. Many studies have confirmed that DM can improve the quality of life or outcomes of treatment, ensure patient satisfaction and control costs [6] by empowering patients through education to better manage their own illness.

Diabetes is prevalent in Louisiana, and management of the disease is challenging due to large low-income populations who may not follow guidelines strictly [7]. According to most studies, patients with type 2 diabetes receive better quality of care from primary care providers than those who seek services in ED. The Health Care Services Division (HSCD) of Louisiana State University is a key provider of care to the uninsured and Medicaid populations in Louisiana. LSU HCSD has implemented a comprehensive, evidence-based, diabetes disease management program to continuously improve diabetic care processes. In the diabetes management program, the components include evidence-based guidelines for physicians and establishment of actionable patient goals with education, medication and clinical support to improve patients' diabetes situations.

When diabetic patients choose the ED for their care, not only do they pay more with no guarantee of appropriate quality services (especially in non-urgent situations) [8-11], but also the health system consumes more resources. From the perspective of disease management, patients with less-urgent conditions in the ED can seek primary care instead. The primary objective of this study is to examine the likelihood that patients who are adhering to the DM program's clinic visit schedules will use the ED for less-urgent problems and conditions.

## Methods

### Population and setting

The primary data source is the Health Care Services Division Disease Management Evaluation Database (DMED), created to monitor patients enrolled in several initiatives of LSU HCSD's disease management programs. The study population was extracted from all 89,567 LSU HCSD diabetes patients with a diagnosis code ICD-9: 250.xx seen in one of the HCSD's eight hospital EDs between 1998 and 2006. After excluding patients with type 1 and other types of diabetics, patients with only one visit, prisoners, and some cases with obvious errors, the resulting data set contained 30,097 type 2 diabetic (ICD-9: 250.x0 and 250.x2) patients with two or more visits in the study period.

Type 2 diabetes patients (n = 30,097) consisted of three groups: the 6.5% of patients who used only emergency department services; the 8.8% who used only the LSU HSCD DM clinics; and 84.6% of patients (n = 25,475) who used both the DM clinics and the ED sites of care. We kept only the last group of patients for this study. Within the study group, we eliminated an additional 10,176 patients who received some type of care in 1998 because we did not know when their DM treatment was initiated. Only the 8,596 patients whose first records appeared in 1999 were retained for the study.

The 8,596 patients in the study group had a total of 220,719 clinic visits and 60,189 ED visits between 1999 and 2006. The ED visits were classified as urgent and less-urgent, based on the ICD-9 codes and review by two nurses who both agreed on the less-urgent ED classification (n = 28,5440) for the visit. Patient ED visits that occurred before the patient's first diabetes-related visit (chief complaint diagnosis codes, ICD-9: 250 to 250.93) were not counted, as well as ED visits that occurred on weekends because the clinics were not open. We kept only data from 6,412 patients who were 45 years and older. The resultant data set contained 119,695 outpatient clinic visits and 16,249 less-urgent ED visits after the first diabetes-related visit occurring on weekdays. This study was approved by Tulane University's Institutional Review Board (IRB#C0344).

### Measures

After removing all urgent ED visits, type 2 diabetic patient visits were classified as less-urgent ED (Y = 1) or clinic visits (Y = 0). Ten independent variables were used in the analyses based on other studies [12,13] including patient age, health plan, duration in the DM program, and facility size where services were received. For the analysis, facilities were classified as large versus small size based on the facility beds (over 100 or below 100 beds). Other variables included the Charlson Comorbidity Index (CCI); a normal A1c rate over the past 12 months based on laboratory test results (normal A1c rate); 12 month adherence to clinic schedules (adherence rate); and the experience of a prior year hospitalization.

Patients with a range of comorbid conditions had each condition assigned a score from 1, 2, 3 and 6 based on Charlson's study [14]. A higher final score means more or more severe comorbidities. We then summed each patient's scores and assigned a total score to represent his/her comorbid conditions as CCI. The observed A1c test results were grouped into three levels (<7% normal, 7-9% borderline and > = 9% high) to calculate the index of normal A1c rate. (= Σ normal level/(Σ normal level + Σ borderline level + Σ high level) in past 12 months).

Adherence to annual patient diabetes-related clinic visit schedules was based on American Diabetes Association suggestions [15], which were separated into three levels of adherence: none (1 point), midpoint (2 points), and high (3 points). For instance, patients who had no diabetes-related clinic visits within the past 12 months got 1 point; patients with 1 or 2 visits, and where the time between the first and second visit was less than 6 months got 2 points; patients with at least 2 visits, where one of the visit periods was longer than 6 months, received 3 points.

### Data Analysis

This study is a longitudinal, retrospective analysis of clinic and less-urgent ED visits from 1999 to 2006. Generalized Estimating Equation (GEE) regression methods for binary responses are appropriate to analyze longitudinal data, especially models with time-dependent variables and repeated measures on the same case. GEE methodology examines the relationship between the occurrence of less-urgent ED visits as compared with clinic visits based on a number of predisposing and enabling factors. The GEE regression model for binary responses identifies those factors that can be altered to reduce the unnecessary use of the ED by diabetic patients participating in the LSU HCSD diabetes disease management program. First, we analyzed the relationship between the outcome and each single unadjusted effect. Then, we computed a full model with all predictors to assess the adjusted variables. All analyses were conducted using SAS 9.12 (SAS Institute, Cary, NC).

## Results

Table 1 and 2 display the descriptive statistics associated with each type of visit. Since the LSU HCSD provides care to uninsured citizens of Louisiana, uninsured patients make up the majority of both less-urgent ED visits and clinic visits, at 69.74% and 66.68% respectively. Most ED and clinic visits were not associated with a serious comorbid condition (CCI = 0). Prior hospitalization over the past 12 months was slightly higher among those who used the clinic rather than the ED; maintaining a normal A1c level over the past 12 months was also slightly greater for patients who sought care in the clinic; and finally clinic visits were more likely to be used by patients who had been more compliant with their DM schedules in the past 12 months.

**Table 1:** Patient Characteristics

Source		Clinic Visits		Less-urgent ED visits	
		N = 119,695		N = 16,249	
		freq	%	freq	%
Health	Commercial	5580	4.66%	1425	8.77%
plan	Medicaid	10143	8.47%	1737	10.69%
	Medicare	20192	16.87%	2136	13.15%
	Missing	307	0.26%	116	0.71%
	Uninsured	83473	69.74%	10835	66.68%

CCI	0	79829	66.69%	11634	71.60%
	1	22432	18.7%	2957	18.20%
	2	11584	9.68%	1138	7.00%
	3	3584	2.99%	303	1.86%
	4+	2266	1.89%	217	1.34%

Hospitalization		24061	20.10%	3088	19.00%

CCI: Charlson Comorbidity Index

**Table 2:** The distribution of risk factors by visits

Source	Clinic Visits			Less-urgent ED visits		
	Mean	SD	N	Mean	SD	N
A1c normal rate*	0.4069	0.44665	79637	0.3536	0.44855	6397
Clinic adherence*	1.34	0.717	105090	1.02	0.181	11386
Duration	3.0005	1.87428	119695	2.7550	1.98243	16249

Note: 1. *those indicators are based on past 12 months period from every visit

2. study period: 1998 - 2006

As seen in the full model in Table 3, age (being older), having a normal A1c level, good adherence rates to the clinic schedule, and duration in the program reduced the likelihood of a less-urgent ED visit. Odds ratios (OR) were 0.9907 (CI: 0.9852 - 0.9962, p = 0.0008) for age; 0.8173 (CI: 0.7444 - 0.8974, p < 0.0002) for a normal A1c rate; 0.1585 (CI: 0.1260 - 0.1994, p < 0.0001) for adherence to clinic schedule in the past 12 months; and 0.9684 (CI: 0.9405 - 0.9907, P = 0.0054) for duration in the program. Being Caucasian or being of another race decreased the likelihood of a less-urgent ED visit as compared with being African-American. ORs were 0.7536 (CI: 0.6880 - 0.8253, p < 0.0001); and 0.7914 (CI: 0.6165 - 0.9998, p = 0.0498), respectively.

**Table 3:** Multi-variable model estimates of the probability of less-urgent ED visits (adjusted for repeat visits)

Whole Model	Exp(b)	95% CI	P
Intercept			
Age (older)*	0.9907	0.9852 0.9962	0.0008
Gender			
Female	Reference		0.2035
Male	1.0619	0.9684 1.1645	
Race			
Black	Reference		<.0001
White*	0.7536	0.6880 0.8253	
Others*	0.7914	0.6265 0.9998	
HealthPlan			
uninsured	Reference		<.0001
Medicaid*	1.2858	1.1165 1.4807	
Medicare*	1.1334	1.0041 1.2793	
Commercial*	1.6958	1.4796 1.9435	
CCI	1.0109	0.9724 1.0509	0.5863
A1c Lab normal*	0.8173	0.7444 0.8974	<.0001
Clinic adherence*	0.1585	0.1260 0.1994	<.0001
Duration in the prog.*	0.9684	0.9465 0.9907	0.0054
Prior hospitalization	1.0414	0.9485 1.1435	0.3945
Facility			
Smaller	Reference		0.1180
Larger	0.9268	0.8427 1.0193	

* is significant, P < 0.05

CCI: Charlosn Comorbidity Index

Note: Estimates from a GEE model that accounts for repeat visits by the same patient

Patients with insurance coverage at the time of the visit had an increased likelihood of a less-urgent ED visit, ranging from 1.13 for being covered by Medicare, and 1.29 for Medicaid enrolees, to 1.70 for commercial insurance coverage as compared with those who were uninsured. Gender, comorbidity, experience of hospitalization in the prior year, and facility size were not statistically significant in predicting the odds that a patient would seek less-urgent care in the ED.

## Discussion

Diabetes DM programs are intended to improve health status and quality of life for type 2 diabetics and reduce costs by educating patients about the benefits of going to clinics and monitoring their disease conditions regularly [16,17]. Other benefits arise from a successful DM program--namely patients who adhere to their DM program [18] or effectively manage their A1c reduce the likely usage of the ED care. Patients go to clinics for diabetes not only for check-ups but also to monitor their diabetes condition through foot and eye exams and to receive updated information to reduce the risk of complications.

Most studies agree that A1c is an important indicator of patient diabetes control. From this study, patients who maintain their A1c at closer to normal levels reduce their likelihood of less-urgent ED visits about 1.22 times. Further, patients who remain in the program longer have a decreased likelihood of less-urgent ED visits even though the effects are all slight. The reason is that, after checking the ED visits' distribution, the most less-urgent emergency situations usually occur during the patient's early visits, such as during the first or second year of the DM program. Aggressive case management to maintain patients' adherence and enhance communication with patients, especially in the first year, may reduce the possibility of future inappropriate ED visits.

In addition, most studies indicate differences in the prevalence of diagnosed diabetes between racial groups. African-Americans are more likely to have diabetes, experienced more complications and face unfavourable prognoses. In this study, African-Americans tended to use more ED services in less-urgent situations than other patients after controlling for insurance coverage, adherence and management of A1c levels. One possible reason may be that they lack access to sufficient primary care resources, resulting in use of less appropriate care from an ED, which may affect their diabetes outcomes.

The Charlson comorbidity index (CCI) did not provide significant information on ED visit use in this study. One reason is that patient visits associated with severe health conditions were more likely to be classified as urgent and thus dropped from the study [19,20].

This study also triggered some interesting questions for future investigations in diabetes research. For example, according to other studies, uninsured patients are more likely to use the ED for care [21,22]. In this study, the uninsured were less likely to seek care in the emergency department for less-urgent situations. A recent study also found that the uninsured do not use the ED more often [23]. We also examined the effect of a patient changing health plans during the study period on the likelihood of less-urgent ED visits, but this variable did not reveal any significant difference.

One of the reasons for patients with health coverage to use the ED for less-urgent visits may be the need to use after-hour services as well as the inability to get timely appointments at the clinic [24,25]. In this study, we controlled for visits over the weekend when the clinics were closed, but could not control for evening visits during the week. Another reason that those with insurance coverage are more likely to use the ED for less-urgent visits may the effect of moral hazard on use. Health insurance reduces the out-of-pocket costs of seeking care in the ED. Some of uninsured may be held liable for the costs of the ED visit if they are not considered indigent [26]. Further study is required to examine the ED usage by insurance status to understand which strategy is suitable to implement in the future.

It seems likely that less-urgent ED visits can be reduced by improving efficiency in primary care by providing a call centre to arrange appointments or case managers to help patients better manage their conditions.

This study used rigid criteria to select the target study population. However, several limitations need to be mentioned. First, we used secondary data and some restrictions, such as the method of data collection, and the coding system, could not be changed. The second limitation is the data time frame. DMED established the data collection system in 1998, and we only selected patients who had medical records from 1999. However, this did not mean these DM programs across the state started at the same time, and some programs were in operation earlier than the start of DMED's implementation. In other words, some patients may have gone to LSU-HCSD (or to another provider for diabetes treatment) before 1998, which may have affected their behaviour during the period of the study. Additionally, some patients may have received some clinical procedures prior to the start date in this study or obtained care in other non-HCSD facilities. There was no information on utilization outside of the HCSD system to enable exclusion of these patients from this study. The third limitation is the behaviour of patients who were only in the DM program for a short duration -- less than one year. Patient attrition of non compliant patients may affect the likelihood of non-urgent ED visits when comparing those who have longer durations of care. The final limitation is a generalized application restriction. These research results apply only to the public system of care and may not be suitable for other health care organizations.

## Conclusion

In this study, we assessed the factors that predispose or enable less-urgent ED visit use among a group of Type 2 diabetes patients seen within a disease management program. Our study provides an example in a natural environment rather than using a randomized controlled trial. The information from this study can help managers adopt strategies to reduce improper patient use of emergency services. Disease managers need to continually improve the clinical protocol that encourages patients to follow the guidelines for clinic visits and obtain regular laboratory tests and examinations. In particular, patients need to remain in their diabetes disease management programs over several years. Adherence to clinic schedules, including guidelines, is still the best way to reduce the likelihood of less-urgent ED visits. We suggest providing reminders for clinic visits, creating continuous care by calling patients regularly or using case managers to reduce the likelihood of less-urgent ED visits. The primary outcome from this investigation provides important information to identify the specific populations who are more likely to use less-urgent ED services. It also provides useful long-term suggestions for reducing the use of ED services while improving quality.

## Competing interests

The authors declare that they have no competing interests.

## Authors' contributions

SCJ and CC jointly drafted every version of the manuscript. RH, LM and RC participated in the design and analysis of the study. RH supervised in acquisition of data. LM provided the statistical consultation. All authors contributed to the discussion and to reviewing the manuscript. All authors read and approved the final manuscript.

## Pre-publication history

The pre-publication history for this paper can be accessed here:

http://www.biomedcentral.com/1472-6963/9/223/prepub
